# Geographic Differences in Public Opinion on Drug Policy: Understanding Patterns of Support and ‘Don't Know’ Responses

**DOI:** 10.1111/dar.70023

**Published:** 2025-08-27

**Authors:** Paul Kelaita, Keelin O'Reilly, Alison Ritter

**Affiliations:** ^1^ Drug Policy Modelling Program, Social Policy Research Centre UNSW Sydney Sydney Australia

**Keywords:** geography, Greater Western Sydney, public opinion, ‘don't know’, surveys

## Abstract

**Introduction:**

Policymakers and advocates often rely on public opinion to support or oppose certain policies, with national surveys providing an important data source. Different geographic areas have socio‐political specificity and are impacted by drug policies in different ways; yet there has been little analysis of public opinion accounting for geographic specificity. This study aimed to understand geographic differences in policy preferences using the case study of Greater Western Sydney (GWS), New South Wales (NSW), Australia.

**Methods:**

Responses to two policy perception questions from Australia's 2022/23 National Drug Strategy Household Survey were analysed: preferred actions in response to possession and use of cannabis, ecstasy, heroin, methamphetamine, and hallucinogens (‘Action’ question), and the preferred criminal offence status of cannabis possession (‘Criminal’ question). Responses in GWS were compared to Rest of Sydney and Rest of NSW. Data were analysed by levels of support and proportions of respondents who did not register an opinion (not answered, ‘don't know’).

**Results:**

Levels of support for different actions and policy settings varied by area, as did the proportions of respondents who do not register an opinion. The inclusion or exclusion of ‘don't know’/not answered responses influences interpretations of levels of public knowledge, engagement, and support.

**Discussion and Conclusion:**

Public opinion as gauged through national surveys should be understood relative to specific geographies. Understanding whether geographic differences exist is important to understanding what drives support and ‘don't know’/not answered responses in relation to drug policy. Differences indicate sites for further analysis and attention in education, engagement and advocacy.

**Summary**

- Depending on context, use of public opinion data that are not differentiated by geography may suggest homogenous levels of policy support and engagement despite much heterogeneity.
- Including or excluding people who ‘did not know’ or did not answer has effects on interpretations of levels of public knowledge, engagement and support for preferred actions and removal of criminal penalties in and between different areas.
- Preferred actions for personal possession and use were no action for cannabis, referral to education for ecstasy, referral to treatment for heroin, methamphetamine and hallucinogens; and the removal of criminal penalties for cannabis in Greater Western Sydney, Rest of Sydney and Rest of New South Wales; however, support for these was lower in Greater Western Sydney.
- There were high proportions of respondents who did not register an opinion when asked which action is preferred regarding personal use and whether personal use of cannabis should be a criminal offence in Greater Western Sydney, Rest of Sydney and Rest of New South Wales; and there were higher proportions of these respondents in Greater Western Sydney.

## 1 Introduction

Public opinion is an important element impacting policy [1, 2, 3, 4]. The impacts are, however, not a simple one‐to‐one calculation of political responsiveness, and instead shift depending on issue salience and institutional context, as well as a range of other policy inputs such as media and political framing [2, 3, 4, 5, 6, 7, 8]. In addition, policy change itself may play a role in shifting public opinion [4, 9, 10]; and how opinion, policy, and the relationship between them are measured [2, 5, 7] all impact what can be said about any kind of causation or association [5, 7]. Furthermore, whose opinion actually influences policy is open to debate, with structural distributions of advantage and disadvantage shaping responsiveness [11]. However, the relationship between public opinion and policy endures and is often tied to questions of democracy and representation [6, 11]. In drug policy, the use of representative datasets, often from national surveys, is a common way in which public attitudes are purportedly captured and represented, and can inform public and policy debate [12, 13, 14, 15]; are analysed by researchers looking to understand how different demographics, social status, or other social characteristics impact perceptions [16, 17, 18, 19]; and how attitudes change over time [19, 20, 21]; and are used by peak and advocacy organisations to support their positions [22, 23, 24].

Just as the opinion‐policy link [5] is not self‐evident, how surveys relate to public opinion depends on what is captured and how those data are framed. Surveys designed to capture public opinion often include the option of ‘don't know’ or for the respondent to not answer. How these responses are analysed may have substantial impacts on how public opinion [25] or level of knowledge [26] are understood and subsequently factor into policy development and change. ‘Don't know’ responses may relate to question design (complexity, length) [27], and may be related to knowledge, understanding, ambivalence and social desirability, among other factors [25]. Purdam et al. [25] note that ‘don't know’ responses to factual questions (where there is a correct answer) relate to knowledge (lack, or not wanting to reveal their knowledge); Purdam et al. [25] also argue that responses to value‐ or attitude‐based questions are likely connected to ‘being uncertain, ambivalent, indifferent or not wanting to answer’. This second category is much more relevant for exploring the role and impact of ‘don't knows’ when it comes to policy attitudes, which have no ‘right answer.’ Differences between country and cross‐national levels of ‘don't know’ responses [25], and in the nature of ‘ambivalence’ [28] suggest that geographic scales are an important analytic frame. Understanding whether subnational and more granular geographic differences exist between public attitudes, and between levels of ‘don't know’ responses is an important first step to exploring the textures of what drives support and ‘don't know’ responses in relation to drug policy. In Australia, publicly released national survey data related to drugs excludes people who select ‘don't know’ or did not answer from the public opinion summary data, which results in the exclusion of this group from public reporting and debate.

This paper aims to answer three research questions: (i) does support for policy responses to drug use and possession for personal use differ between geographic areas?; (ii) are there different proportions of respondents selecting ‘don't know’ or who did not answer in different geographic areas?; and (iii) do proportions of ‘don't know’/not answered influence how levels of support for policy responses are understood? To answer these questions, we use the case study of Greater Western Sydney (GWS), with comparison to responses in the Rest of Sydney and New South Wales (NSW), Australia.

### 1.1 Case Study

Drug policy covers drug laws, treatment, prevention, harm reduction and supply reduction. This case study focuses on public opinion toward drug laws, and more specifically whether drug use should be a criminal offence and what actions should be taken in response. In Australia, despite a National Drug Strategy, each state/territory determines their policy priorities and drug laws. While all states and territories have what may be understood as decriminalised or diversionary policies with regard to the personal possession and use of illicit drugs [29] there are substantial state differences. In NSW, where our case study is located, there are diversionary schemes for cannabis and other illicit drugs, with both retaining criminal offences and penalties for possession and use. The cannabis scheme (‘Cannabis Cautioning Scheme’, 2001) involves a caution and voluntary referral to education; the illicit scheme (‘Early Drug Diversion Initiative’, 2024) involves a fine ($400) or referral to a health and education session.

While any area might have been chosen to answer our research questions, we have chosen to focus on GWS, which is a large and populous geographic region of Greater Sydney. GWS (and particular subregions, e.g., Western Sydney, South‐west Sydney) is an area in Sydney and NSW generally understood as a place that has historically been the home of working class communities and is now primarily understood as a place of ethnic diversity with large East and Southeast Asian, Middle Eastern, Pasifika and African communities [30, 31, 32, 33, 34, 35]. Lessons drawn from other areas of public policy (e.g., same‐sex marriage) indicate that Western Sydney is a politically and culturally charged site imagined as an area seemingly at odds with mainstream cultural attitudes [32] and seen as a political battleground for elections [36]. The political importance of this area is underscored by the high number of voters and strategically significant seats in both state and federal politics [36, 37].

Data related to the drug diversion schemes described above, and studies looking at areas or populations in GWS show that experiences of drugs and drug policy in GWS are shaped by policy implementation, geographic dislocation, and stigma unique to the area. Despite being state‐wide policy, the cannabis scheme has seen different geographic effects due to policing practice [38] and uneven application in areas such as GWS [39]. Additionally, specific groups such as culturally and linguistically diverse and First Nations peoples are disproportionally impacted by uneven and discriminatory policing practices [40] and policy design [41], with large culturally and linguistically diverse and First Nations communities in GWS. The illicit drug diversion scheme also had, in its first 6 months of operation in 2024, different diversion rates for Indigenous people, drug types, and across different areas of the state, with areas in GWS having diversion rates of between 0% and 32% [42].

Studies looking at areas or populations in GWS and drugs have focused on patterns of drug use and risk [43, 44, 45, 46, 47, 48, 49, 50, 51, 52], rates of overdose deaths [53], the politics and accessibility of supervised drug consumption facilities [53, 54], needle syringe programs [55, 56], policing [57, 58, 59, 60, 61], and stigma and discrimination [62, 63]. In these studies, drug use and drug practices data for GWS (or a subset of GWS) are compared to other places (e.g., analysing differences between people receiving opioid agonist treatment in parts of GWS compared to their inner city counterparts and what may drive different injecting practices) [43], included in broader studies and not foregrounded in analysis [44, 50], or are studied precisely because of identified issues with or perceptions of the area (e.g., suburban expansion, overdose death rates, heroin availability, ethnic communities) [45, 46, 47, 48, 49]. In addition, experiences of drugs, laws, and policing in areas of GWS are connected to the specific physical and social resonances of the area. For example, access to services is shaped by physical distances that often necessitate high‐risk practices which may relate to use itself (e.g., injecting further from health services) or the way a service is accessed (e.g., travelling long distances on public transport to access a supervised injecting site while carrying a personal amount of drugs) [53, 55]; policing practices and perceptions of those practices are shaped by the association of specific areas in GWS with certain ethnic groups [58, 60]; and stigma and discrimination relate to geographic marginalisation and the class and ethnic dimensions described above [51, 62, 63] (e.g., including through pejorative place‐based terms such as “westie” [64]). Whether to do with policy implementation, drug use and risk demographics, or specific social and physical dimensions of geography, there are meaningful differences related to drugs and drug policy when it comes to GWS compared to other areas.

There is no consistent way of representing GWS or its subregions, and yet its distinctiveness in relation to politics, culture and drugs makes it a useful area of focus when thinking about geography, public opinion and drug policy in NSW.

## 2 Method

We analysed the 2022/23 National Drug Strategy Household Survey (NDSHS). Ethics was obtained from UNSW HREC (iRECS7002); unit record data were provided on request through the Australian Data Archive (ADA Dataverse) by the Australian Institute of Health and Welfare.

The NDSHS is an Australian survey conducted every 3 years about drug use and attitudes/perceptions of drugs and drug policy, using a sample of people aged 14 and over [65]. The 2022/2023 wave of the survey contained 21,663 respondents, representing a 43.9% response rate [66]; 72% of respondents completed the survey via paper form, 28% online and 0.1% via telephone [66]. The NDSHS is considered a nationally representative survey but, like most household surveys, excludes people in hospitals, hostels, prisons and other similar institutions, as well as excluding people experiencing homelessness or unstable housing [66]. This means that many people who use drugs, and particularly more marginalised people, are excluded or not adequately represented [67].

**FIGURE 1:**
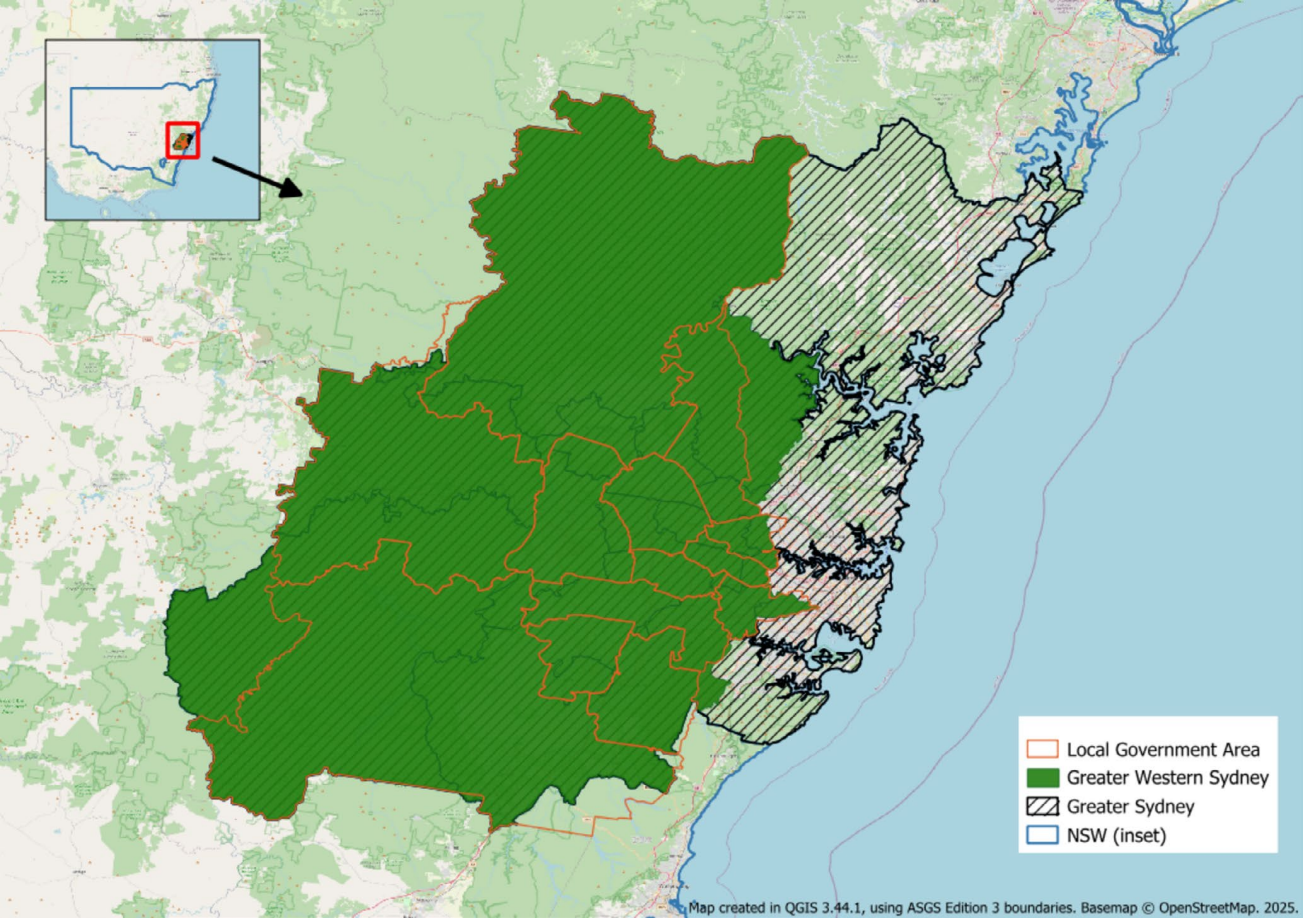
Map of Greater Western Sydney, Greater Sydney and Local Government Area boundaries. *Source*: Map created in QGIS 3.44.1, using Australian Statistical Geography Standard Edition 3 boundaries (available at https://data.gov.au/data/dataset/asgs‐edition‐3‐2021‐boundaries) CC BY 4.0. Local Government Area: Non ABS structure boundaries (2025); Greater Western Sydney: SA3 structure (2021), see Appendix Table A1 for full list of SA3s used; NSW and Greater Sydney boundaries: Greater Capital City Statistical Areas structure (2021). Basemap^©^ OpenStreetMap contributors [79]. 2025.

Geographic classifications used in the NDSHS are based on the Australian Statistical Geography Standard [68]. Data in the NDSHS are collected by statistical area 1 (SA1), which aggregate into larger SA2s, SA3s and so on [68]. GWS is defined in a variety of ways that do not map neatly onto state or federal electorates, or popular imaginaries. We defined GWS using an aggregation of SA3s that approximate the boundaries of well‐known classifications (the 13 local government areas that make up GWS) and less well‐defined cultural imaginaries (which break down GWS around class and race profiles (35); see Appendix Table A1). We report the results by a total consolidated GWS variable that includes all chosen SA3s. As a result, GWS data below approximates GWS as defined around the 13 local government areas (see Figure 1). We compare GWS to data for the rest of Greater Sydney (‘Rest of Sydney’) and the rest of the state of NSW (‘Rest of NSW’). Sydney in the NDSHS data refers to Greater Sydney as defined by the Australian Statistical Geography Standard Greater Capital City Statistical Areas [68] (see Figure 1). We remove the region identified by our GWS variable from Greater Sydney and the state of NSW to define Rest of Sydney and Rest of NSW.

**FIGURE 2:**
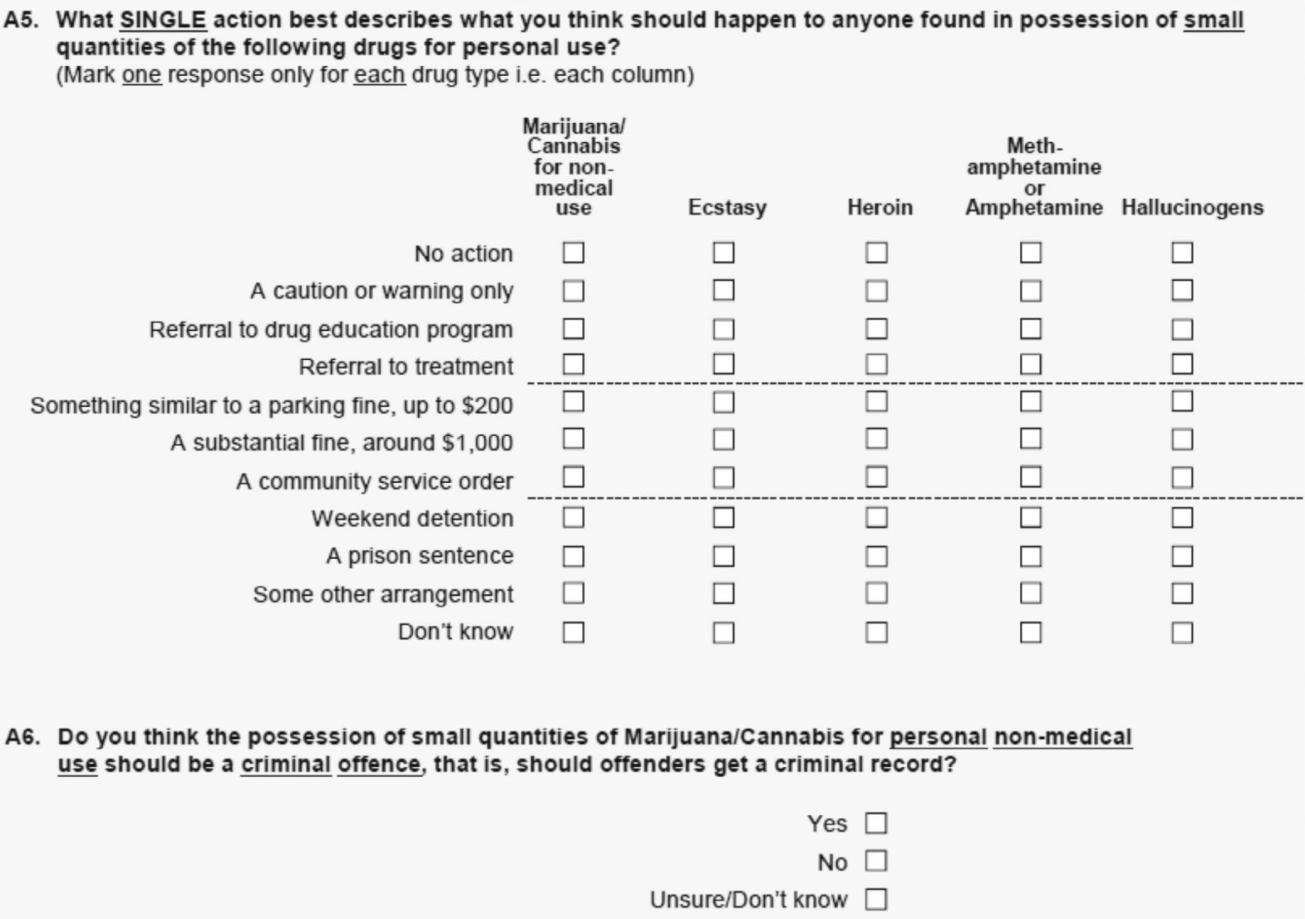
National Drug Strategy Household Survey 2022–2023, questions A5, A6. *Source*: Reference [80].

Survey responses to two questions are analysed; see Figure 2. We refer to A5 as the ‘Action’ question, and to A6 as the ‘Criminal’ question. These two questions were chosen to compare levels of support for specific actions, support for the removal of criminal penalties, and the levels and impact of ‘don't know’ and unanswered responses.

We applied the person‐level weights provided by the Australian Institute of Health and Welfare (‘absolute person weight’) which takes into account the survey design by scaling the survey sample to reflect population characteristics (from the Australian Bureau of Statistics census data, e.g., age, sex). We scaled these person‐level weights to the effective sample size as calculated by Roy Morgan [69] (for NSW total, not specific to GWS) to account for the overinflation of the sample resulting from the person‐level weights. The new weight (based on the effective sample size) was calculated and applied using SPSS (v27). Table 1 shows the actual survey sample size and the effective sample size for each of our regions as well as Greater Sydney and NSW total. All analyses reported are calculated using the effective sample size weight.

**TABLE 1:** Survey sample size and effective sample size of each region.

Region	Survey sample size (*n*)	Effective sample size (*n*)
Greater Western Sydney	1589	1103
Rest of Sydney	2106	1106
Rest of NSW	3985	2309
Greater Sydney total	3695	2210
**NSW total**	**5574**	**3412**

*Note*: The effective sample size is lower than the survey sample size as it takes into account the survey method (see [69] for more details).

Abbreviation: NSW, New South Wales.

Analyses were undertaken using SPSS (v27). Cross‐tabulations (with 95% confidence intervals, calculated in Excel) were used to analyse the level of support for both questions in each region, and Pearson chi‐squared tests of independence were used to compare select responses in GWS to Rest of Sydney and Rest of NSW (statistical significance was evaluated at the 5% level).

We grouped ‘don't know’ responses and those that did not answer as ‘no opinion registered’ to understand differences between areas. We grouped these for two additional reasons: the public release NDSHS data excludes people who select ‘don't know’ or did not answer from their base. The presumption here is that public support is gauged from those who feel informed enough to respond or respond regardless of whether they feel informed. The second is that reasons for answering ‘don't know’ are diverse and do not relate to a single explanation. In survey questions, these kinds of options which fail to register an opinion or attitude have been framed as ‘no opinion’ or ‘non‐attitude’ questions [70]. The NDSHS approach and the broader category of ‘no opinion’ questions suggests that both ‘don't know’ and ‘no answer’ responses should be considered together. Given our third research question, we analysed the data including and excluding these respondents.

## 3 Results

### 3.1 Levels of Support for Policy Responses to Personal Use and Possession

Across the regions and drug types, no single action received a ‘majority’ preference among respondents. The actions with the highest level of support across all three regions were consistent (Table 2; see Appendix Table A2 for full table of responses to actions): no action for cannabis (23.1% in GWS to 39.4% in Rest of Sydney); referral to education for ecstasy (18.1% in GWS to 19.5% in Rest of Sydney and Rest of NSW); and referral to treatment for heroin (25.9% in GWS to 35.1% in Rest of Sydney), methamphetamine (24.9% in GWS to 33.3% in Rest of Sydney) and hallucinogens (18.8% in GWS to 19.3% in Rest of NSW).

**TABLE 2:** Preferred action that should be taken against people found in possession of selected illicit drugs for personal use, by region, 2022/2023.

Top selected actions	Greater Western Sydney, % (95% CI)	Rest of Sydney, % (95% CI)	Rest of NSW, % (95% CI)
**Cannabis**	***n* = 1104**	***n* = 1107**	***n* = 2306**
1.	No action 23.1 (20.6, 25.6)	No action 39.4 (36.5, 42.3)	No action 37.6 (35.6, 39.5)
2.	Caution or warning 18.1 (15.8, 20.4)	Caution or warning 19.0 (16.7, 21.3)	Caution or warning 19.9 (18.3, 21.5)
3.	Referral to education	Referral to education	Referral to education
	14.8 (12.7, 16.9)	13.2 (11.2, 15.2)	14.2 (12.8, 15.6)
4.	Substantial fine^a^ 8.3 (6.7, 10.0)	Small fine^a^ 5.5 (4.2, 6.9)	Small fine^a^ 5.5 (4.5, 6.4)
Total top four actions	64.3 (61.5, 67.1)	77.1 (74.6, 79.5)	77.1 (75.4, 78.8)
**Ecstasy**	***n* = 1102**	***n* = 1106**	***n* = 2309**
1.	Referral to education 18.1 (15.9, 20.4)	Referral to education 19.5 (17.2, 21.9)	Referral to education 19.5 (17.9, 21.1)
2.	Substantial fine 14.6 (12.5, 16.7)	Caution or warning 16.5 (14.3, 18.6)	Caution or warning 15.1 (13.6, 16.5)
3.	Referral to treatment	No action	Referral to treatment
	12.3 (10.4, 14.3)	14.1 (12.1, 16.2)	12.3 (10.9, 13.6)
4.	Don't know^a^ 9.5 (7.8, 11.3)	Referral to treatment^a^ 11.0 (9.2, 12.9)	Substantial fine^a^ 11.2 (9.9, 12.5)
Total top four actions	54.6 (51.7, 57.6)	61.1 (58.2, 64.0)	58.0 (56.0, 60.0)
**Heroin**	***n* = 1103**	***n* = 1106**	***n* = 2309**
1.	Referral to treatment 25.9 (23.3, 28.5)	Referral to treatment 35.1 (32.3, 37.9)	Referral to treatment 32.9 (31.0, 34.8)
2.	Prison 16.7 (14.5, 18.9)	Referral to education 16.5 (14.4, 18.7)	Referral to education 15.6 (14.1, 17.1)
3.	Substantial fine	Prison	Prison
	14.1 (12.0, 16.1)	12.1 (10.2, 14.0)	14.2 (12.7, 15.6)
4.	Referral to education 13.8 (11.7, 15.8)	Substantial fine 9.2 (7.5, 10.9)	Substantial fine 10.6 (9.4, 11.9)
Total top four actions	70.4 (67.8, 73.1)	73.0 (70.3, 75.6)	73.2 (71.4, 75.0)
**Methamphetamine**	***n* = 1103**	***n* = 1105**	***n* = 2308**
1.	Referral to treatment 24.9 (22.4, 27.5)	Referral to treatment 33.3 (30.5, 36.1)	Referral to treatment 31.5 (29.6, 33.4)
2.	Prison 16.1 (14.0, 18.3)	Referral to education 15.5 (13.3, 17.6)	Prison 15.0 (13.6, 16.5)
3.	Substantial fine	Prison	Referral to education
	13.3 (11.3, 15.3)	11.4 (9.5, 13.3)	14.2 (12.8, 15.6)
4.	Referral to education^a^ 13.1 (11.1, 15.0)	Substantial fine^a^ 8.6 (6.9, 10.3)	Substantial fine 9.4 (8.3, 10.6)
Total top four actions	67.5 (64.7, 70.2)	68.8 (66.0, 71.5)	70.2 (68.4, 72.1)
**Hallucinogens**	***n* = 1103**	***n* = 1106**	***n* = 2308**
1.	Referral to treatment 18.8 (16.5, 21.1)	Referral to treatment 18.9 (16.6, 21.2)	Referral to treatment 19.3 (17.7, 20.9)
2.	Referral to education 16.1 (14.0, 18.3)	Referral to education 16.9 (14.7, 19.1)	Referral to education 17.2 (15.7, 18.8)
3.	Don't know	No action	No action
	12.8 (10.8, 14.8)	12.8 (10.9, 14.8)	9.8 (8.6, 11.1)
4.	Prison^a^ 11.5 (9.6, 13.4)	Caution or warning^a^ 10.6 (8.8, 12.4)	Prison^a^ 9.0 (7.8, 10.2)
Total top four actions	59.0 (56.1, 61.9)	59.2 (56.3, 62.1)	55.4 (53.3, 57.4)

*Note*: Base includes respondents who did not answer or selected ‘Don't know’. Question: ‘What *SINGLE* action best describes what you think should happen to anyone found in possession of *small* quantities of the following drugs for personal use?’ (emphasis in original survey). Options: ‘No action’, ‘A caution or warning only’, ‘Referral to drug education program’, ‘Referral to treatment’, ‘Something similar to a parking fine, up to $200’, ‘A substantial fine, around $1000’, ‘A community service order’, ‘Weekend detention’, ‘A prison sentence’, ‘Some other arrangement’, ‘Don't know’.

Abbreviations: CI, confidence interval; NSW, New South Wales.

^a^ CI overlap with responses to the next most selected action.

Despite all three regions returning the same most preferred action for all drug types, the level of support for the preferred actions was lower in GWS compared to the other regions for cannabis (*p* ≤ 0.001), heroin (*p* ≤ 0.001) and methamphetamine (*p* ≤ 0.001). For example, while ‘no action’ was the most preferred response to cannabis in all regions, this was under a quarter (23.1%) in GWS compared to over a third for Rest of Sydney (39.4%, *p* ≤ 0.001) and Rest of NSW (37.6%, *p* ≤ 0.001). There was no evidence of a difference for the most preferred action of GWS compared to Rest of Sydney and Rest of NSW for ecstasy (*p* = 0.395 and *p* = 0.325, respectively) and hallucinogens (*p* = 0.938 and *p* = 0.725, respectively).

Beyond the most preferred action, there are differences in second, third, and so on most selected actions by area and by drug type (see Table 2).

For criminal penalties, the majority of respondents in GWS, Rest of Sydney, and Rest of NSW support the removal of criminal penalties for the personal non‐medical use of cannabis. GWS returned the lowest, though still a majority (52.7% [95% confidence interval (CI) 49.8, 55.7]), support for the removal of criminal penalties for cannabis compared to both Rest of Sydney (70.3% [95% CI 67.6, 72.9], *p* ≤ 0.001) and Rest of NSW (69.4% [95% CI 67.5, 71.3], *p* ≤ 0.001) (see Appendix Table A3 for full table of responses).

### 3.2 Proportions of ‘Don't Know’ and Did Not Answer Responses

As shown in Table 3, there are substantial proportions of respondents registering no opinion (‘don't know’ and did not answer) for both the Action and Criminal questions. In response to the Action questions, ‘don't know’ respondents were lowest for cannabis and highest for hallucinogens across all regions. Combining respondents who ‘don't know’ and did not answer (no opinion registered, see Table 3) shows that for the Action question up to a fifth of respondents (13.4%–21.1%) in GWS did not register an opinion, compared to a seventh of respondents in Rest of Sydney (9.2%–14.4%, *p* values all < 0.005) and NSW (7.8%–14.1%, *p* values all < 0.001). Similarly, in response to the Criminal question, nearly a quarter of respondents (23.6%) in GWS did not register an opinion compared to a sixth of respondents in Rest of Sydney (16.5%, *p* ≤ 0.001) and Rest of NSW (15.3%, *p* ≤ 0.001).

**TABLE 3:** Respondents who did not register an opinion in response to ‘Action’ and ‘Criminal’ question, by region, 2022/2023.

	Greater Western Sydney		Rest of Sydney			Rest of NSW		
	*n*	No opinion registered, % (95% CI)	*n*	No opinion registered, % (95% CI)	*p*	*n*	No opinion registered, % (95% CI)	*p*
‘Action’: Cannabis	**1104**	**13.3 (11.3, 15.3)**	1107	9.2 (7.5, 10.9)	0.003	2306	7.8 (6.8, 8.9)	< 0.001
‘Action’: Ecstasy	**1102**	**17.7 (15.4, 19.9)**	1106	11.9 (10.0, 13.8)	< 0.001	2309	12.0 (10.6, 13.3)	< 0.001
‘Action’: Heroin	**1103**	**14.8 (12.7, 16.9)**	1106	10.4 (8.6, 12.2)	0.002	2309	10.4 (9.2, 11.7)	< 0.001
‘Action’: Methamphetamine	**1103**	**17.6 (15.3, 19.8)**	1105	12.9 (11.0, 14.9)	0.002	2308	12.3 (11.0, 13.7)	< 0.001
‘Action’: Hallucinogens	**1103**	**21.2 (18.7, 23.5)**	1106	14.4 (12.3, 16.4)	< 0.001	2308	14.1 (12.7, 15.5)	< 0.001
‘Criminal’: Cannabis	**1104**	**23.6 (21.0, 26.1)**	1106	16.5 (14.3, 18.6)	< 0.001	2309	15.3 (13.8, 16.8)	< 0.001

*Note*: No opinion registered, includes: ‘Don't know’ and did not answer. *p* Value is based on Pearson chi‐squared test of independence. Bold emphasis for the Greater Western Sydney column indicates that this is the main comparator (i.e., *p* values in the Sydney and NSW columns are tested against GWS).

Abbreviations: CI, confidence interval; NSW, New South Wales.

### 3.3 Impact of ‘Don't Know’/Did Not Answer Responses on Level of Policy Support

Despite the substantial proportions of respondents registering no opinion, excluding ‘don't knows’ and those that did not answer from the base sample for the Action question does not change the ‘most preferred action’ in any of the regions; however, Table 4 shows the considerable difference in the raw level of support when including and excluding those with no opinion registered (see Appendix Table A2 for full table). As expected, excluding these responses results in an increase in support for all actions (e.g., support for referral to treatment for methamphetamine in GWS jumps from 24.9% to 30.3%). Likewise, for the Criminal question, support for removal of criminal penalties for possession of cannabis for personal use in GWS increases from 52.7% (95% CI 49.8, 55.7) to 69% (95% CI, 65.8, 72.1) when excluding those who don't know or did not answer (see Appendix Table A3 for full table).

**TABLE 4:** Level of support for the ‘most preferred action’ when including and excluding those with no opinion registered.

Drug type	Preferred action	Greater Western Sydney, % (95% CI)	Rest of Sydney, % (95% CI)	Rest of NSW, % (95% CI)
**Cannabis**				
Incl no opinion reg	No action	23.1 (20.6, 25.6)	39.4 (36.5, 42.3)	37.6 (35.6, 39.5)
Excl no opinion reg	No action	26.6 (23.8, 29.4)	43.4 (40.3, 46.4)	40.8 (38.7, 42.8)
**Ecstasy**				
Incl no opinion reg	Ref to education	18.1 (15.9, 20.4)	19.5 (17.2, 21.9)	19.5 (17.9, 21.1)
Excl no opinion reg	Ref to education	22.1 (19.4, 24.7)	22.2 (19.6, 24.8)	22.2 (20.4, 24.0)
**Heroin**				
Incl no opinion reg	Ref to treatment	25.9 (23.3, 28.5)	35.1 (32.3, 37.9)	32.9 (31.0, 34.8)
Excl no opinion reg	Ref to treatment	30.4 (27.5, 33.4)	39.2 (36.1, 42.2)	36.7 (34.6, 38.8)
**Methamphetamine**				
Incl no opinion reg	Ref to treatment	24.9 (22.4, 27.5)	33.3 (30.5, 36.1)	31.5 (29.6, 33.4)
Excl no opinion reg	Ref to treatment	30.3 (27.3, 33.2)	38.3 (35.2, 41.3)	36.0 (33.9, 38.1)
**Hallucinogens**				
Incl no opinion reg	Ref to treatment	18.8 (16.5, 21.1)	18.9 (16.6, 21.2)	19.3 (17.7, 20.9)
Excl no opinion reg	Ref to treatment	23.8 (21.0, 26.6)	22.1 (19.4, 24.7)	22.5 (20.6, 24.3)

*Note*: No opinion registered, includes: ‘Don't know’ and did not answer.

Abbreviations: CI, confidence interval; NSW, New South Wales.

The ‘no opinion registered’ grouping also impacts understandings of most and subsequent (second, third etc.) preferred actions. This is most pronounced in GWS, which has the highest proportions of those respondents. In GWS, this ‘no opinion registered’ group was the most selected response for hallucinogens; and equivalent to the most preferred action for ecstasy and the second most selected action for methamphetamine.

## 4 Discussion

The findings of this study illustrate that public opinion on drug policy with regard to actions taken or criminal offence status related to illicit drugs is geographically specific. Depending on context, use of public opinion results that are not differentiated by geography may suggest homogenous levels of policy support and engagement despite much heterogeneity. For our case study of Greater Western Sydney, this study has shown that: (i) there are consistent patterns of support for the most preferred action in response to illicit drugs, and the removal of criminal penalties for cannabis across the geographic regions of GWS, Rest of Sydney and Rest of NSW; however, support is lower in GWS than Rest of Sydney and NSW; (ii) there are higher levels of respondents who ‘don't know’ or did not answer which action is preferred regarding personal use and whether personal use of cannabis should be a criminal offence in GWS than in Rest of Sydney and NSW; and (iii) excluding ‘don't knows’/not answered from analysis may inflate or remove important context from understandings of the specific level of public support, and ignore different levels of drug policy knowledge and engagement in different areas. This study illustrates that there are geographic differences in public support for particular policy settings or actions, and that ‘don't know’ respondents are important elements in interpreting drug policy preference data as they relate to different geographies.

In responses to the Action question, there was no single action that received majority support. Most preferred actions are consistent across the geographic areas of focus. Preferred actions for personal possession and use were no action for cannabis, referral to education for ecstasy, referral to treatment for heroin, methamphetamine, and hallucinogens, and the removal of criminal penalties for cannabis in GWS, Rest of Sydney and Rest of NSW. GWS returned lower levels of support for most preferred actions for ecstasy, heroin, and methamphetamine, and for the removal of criminal penalties for cannabis.

There is a consistently high proportion of respondents registering no opinion in our three geographic areas for both the Action and Criminal questions. Up to one in five respondents in GWS and one in seven in Rest of Sydney and NSW did not register an opinion on preferred actions; and up to one in four in GWS and one in six in Rest of Sydney and NSW did not register an opinion on whether cannabis use should remain a criminal offence. There were higher proportions of those not registering an opinion in GWS compared to both Rest of Sydney and NSW for both questions and across all drug types. The high proportion of people who did not register an opinion, particularly in GWS, suggests that people who do not know or do not answer represent an important site of further analysis and attention in education, engagement, and advocacy. More attention to geography and how it relates to substantive opinions on responses to different drugs should be taken into consideration when assessing levels of support for specific actions. Given that ‘knowledge’ may be only one reason why people ‘don't know’ [25], and that policy preferences are attitudinal rather than factual, there is scope for disseminating information around the evidence base for different policies. This is also the case for the higher levels of support for more punitive, criminalised mechanisms, which have evidence of substantial harms [71, 72, 73, 74, 75]. However, given different reasons underpinning ‘don't know’ responses [25], that the media plays a role in shaping public opinion [76], and that factual statements about particular policies do not necessarily alone increase public support [77, 78], there is also scope for engaging those who may not know in different ways to address other barriers such as understanding, ambivalence or perceived social desirability issues.

The high proportions of respondents not registering an opinion factor into understanding levels of policy support in two ways in the current analysis. The first is that excluding those respondents may inflate understandings of the specific level of public support for particular policies, in this case by a factor between 1.1 to 1.2 (actions taken) and 1.2 to 1.3 (criminal penalties) times. The second is that assessing respondents who do not register an opinion as a group tempers understandings of the preferred actions; that group in GWS for hallucinogens, for example, represents a bigger proportion of respondents than any single action and for ecstasy, the same proportion as the more preferred action. While the logic underpinning the exclusion of these respondents in representations of public attitudes is to gauge the strength of substantive attitudes toward an approach or issue, their omission also loses the opportunity to understand other parts of the drug policy picture, including how issue salience or engagement and knowledge of drug policy change in different areas.

Limitations of this study include the data source, geographic scale, and focus of our case study. There may be other ways to gauge public support for policy responses that do not rely on household surveys. The NDSHS has sampling limits discussed in the method section, including exclusion of unhoused people and people in institutions (e.g., prisons). Stigma around drug use may also limit the data when it comes to representing people who use drugs, and may have specific social desirability impacts when answering policy support questions. As noted above, this may also impact respondents returning ‘don't know’ or not answering items. We could have chosen any geographic sub‐unit for this study (as well as different geographic boundaries for GWS given there is no agreed definition for this subregion), and the extent to which ‘no opinion registered’ responses impact levels of support across other geographic areas remains unknown. Likewise, we could have chosen policy topics other than criminal penalties, and it remains to be seen how ‘don't know’ and no answer responses impact opinions on other drug policy topics. Additionally, this study did not aim to examine whether current context, politics, culture, or drug policy implementation drives the opinions or the ‘don't know’ responses discussed; that is a fruitful area for future research.

## 5 Conclusion

Public support for drug policies is an important part of the picture of policy debate and change. Interpretation of public opinion is dependent on how data are understood to relate to different geographies. The high proportions of respondents who do not know or do not answer, and in particular the way these proportions differ between geographic areas, should be specified to more accurately convey the benefits and limitations of survey data to drug policy debate. There is scope for specific interventions to increase engagement, to explore different participatory processes, and/or political and other public leadership in areas of drug policy that have particularly high proportions of people who do not register an opinion.

## Author Contributions

Each author certifies that their contribution to this work meets the standards of the International Committee of Medical Journal Editors.

## Conflicts of Interest

The authors declare no conflicts of interest.

## Data Availability

The data that support the findings of this study are available from Australian Data Archive. Restrictions apply to the availability of these data, which were used under license for this study. Data are available from https://dataverse.ada.edu.au/dataverse/ada with the permission of Australian Data Archive.

## Appendix A

**TABLE A1:** Greater Western Sydney, by aggregated SA3 across four regions.

Group name		SA3 name	SA3 code
Greater Western Sydney	Central Western Sydney	Canterbury	11,902
		Auburn	12,501
		Merrylands—Guildford	12,503
		Parramatta	12,504
		Blacktown	11,601
		Bankstown	11,901
		Penrith	12,403
		Mount Druitt	11,603
		St Mary's	12,405
	Outer Western Sydney	Blue mountains—south	12,402
		Blue mountains	12,401
	South Western Sydney	Fairfield	12,702
		Campbelltown	12,302
		Liverpool	12,703
		Camden	12,301
		Wollondilly	12,303
		Bringelly—Green Valley	12,701
	North Western Sydney	Richmond—Windsor	12,404
		Hawkesbury	11,503
		Dural—Wisemans Ferry	11,502
		Baulkham Hills	11,501
		Blacktown—North	11,602
		Carlingford	12,502
		Rouse Hill—McGraths Hills	11,504

**TABLE A2:** Preferred action that should be taken against people found in possession of selected illicit drugs for personal use, by region, 2022/2023.

	Greater Western Sydney (incl. don't know/not answered) (%)	Greater Western Sydney (excl. don't know/not answered) (%)	Rest of Sydney (incl. don't know/not answered) (%)	Rest of Sydney (excl. don't know/not answered) (%)	Rest of NSW (incl. don't know/not answered) (%)	Rest of NSW (excl. don't know/not answered) (%)
Cannabis						
*n*	1104	957	1107	1005	2306	2125
No action	23.1 (20.6, 25.6)	26.6 (23.8, 29.4)	39.4 (36.5, 42.3)	43.4 (40.3, 46.4)	37.6 (35.6, 39.5)	40.8 (38.7, 42.8)
Caution or warning	18.1 (15.8, 20.4)	20.9 (18.3, 23.5)	19.0 (16.7, 21.3)	20.9 (18.4, 23.4)	19.9 (18.3, 21.5)	21.6 (19.9, 23.3)
Referral—education	14.8 (12.7, 16.9)	17.0 (14.7, 19.4)	13.2 (11.2, 15.2)	14.5 (12.3, 16.7)	14.2 (12.8, 15.6)	15.4 (13.9, 16.9)
Referral—treatment	7.2 (5.7, 8.8)	8.4 (6.6, 10.1)	5.0 (3.7, 6.2)	5.5 (4.1, 6.9)	5.3 (4.4, 6.2)	5.7 (4.8, 6.7)
Fine $200	5.7 (4.3, 7.1)	6.6 (5.0, 8.2)	5.5 (4.2, 6.9)	6.1 (4.6, 7.5)	5.5 (4.5, 6.4)	5.9 (4.9, 6.9)
Fine $1000	8.3 (6.7, 10.0)	9.6 (7.7, 11.5)	4.1 (2.9, 5.2)	4.5 (3.2, 5.8)	4.7 (3.8, 5.5)	5.1 (4.1, 6.0)
Community service order	3.0 (2.0, 4.0)	3.4 (2.3, 4.6)	1.5 (0.8, 2.3)	1.7 (0.9, 2.5)	1.8 (1.3, 2.4)	2.0 (1.4, 2.6)
Weekend detention	0.9 (0.3, 1.5)	1.0 (0.4, 1.7)	0.7 (0.2, 1.2)	0.8 (0.2, 1.3)	0.7 (0.3, 1.0)	0.7 (0.3, 1.1)
Prison	4.6 (3.4, 5.9)	5.3 (3.9, 6.8)	2.3 (1.4, 3.1)	2.5 (1.5, 3.5)	2.3 (1.7, 2.9)	2.5 (1.8, 3.2)
Other	0.9 (0.3, 1.5)	1.0 (0.4, 1.7)	0.2 (−0.1, 0.4)	0.2 (−0.1, 0.5)	0.3 (0.1, 0.5)	0.3 (0.1, 0.6)
Don't know	8.0 (6.4, 9.6)		5.4 (4.1, 6.8)		4.6 (3.7, 5.4)	
Not answered	5.3 (4.0, 6.7)		3.8 (2.7, 4.9)		3.3 (2.6, 4.0)	
Total	100	100	100	100	100	100
Ecstasy						
*n*	1102	907	1106	974	2309	2933
No action	3.9 (2.8, 5.0)	4.7 (3.4, 6.1)	14.1 (12.1, 16.2)	16.0 (13.7, 18.3)	10.5 (9.2, 11.7)	11.9 (10.5, 13.3)
Caution or warning	9.3 (7.6, 11.1)	11.4 (9.3, 13.4)	16.5 (14.3, 18.6)	18.7 (16.2, 21.1)	15.1 (13.6, 16.5)	17.1 (15.5, 18.8)
Referral—education	18.1 (15.9, 20.4)	22.1 (19.4, 24.7)	19.5 (17.2, 21.9)	22.2 (19.6, 24.8)	19.5 (17.9, 21.1)	22.2 (20.4, 24.0)
Referral—treatment	12.3 (10.4, 14.3)	15.0 (12.7, 17.3)	11.0 (9.2, 12.9)	12.5 (10.4, 14.6)	12.3 (10.9, 13.6)	13.9 (12.4, 15.4)
Fine $200	6.9 (5.4, 8.4)	8.4 (6.6, 10.2)	5.8 (4.4, 7.2)	6.6 (5.0, 8.1)	5.6 (4.7, 6.6)	6.4 (5.3, 7.5)
Fine $1000	14.6 (12.5, 16.7)	17.8 (15.3, 20.2)	10.0 (8.3, 11.8)	11.4 (9.4, 13.4)	11.2 (9.9, 12.5)	12.7 (11.2, 14.1)
Community service order	3.0 (2.0, 4.0)	3.6 (2.4, 4.9)	3.5 (2.4, 4.6)	4.0 (2.8, 5.2)	3.5 (2.7, 4.2)	3.9 (3.1, 4.8)
Weekend detention	3.5 (2.4, 4.6)	4.3 (3.0, 5.6)	1.7 (1.0, 2.5)	2.0 (1.1, 2.8)	2.6 (2.0, 3.3)	3.0 (2.3, 3.7)
Prison	9.4 (7.7, 11.2)	11.5 (9.4, 13.5)	5.2 (3.9, 6.6)	6.0 (4.5, 7.4)	7.0 (5.9, 8.0)	7.9 (6.7, 9.1)
Other	1.1 (0.5, 1.7)	1.3 (0.6, 2.1)	0.6 (0.2, 1.1)	0.7 (0.2, 1.2)	0.8 (0.5, 1.2)	0.9 (0.5, 1.4)
Don't know	9.5 (7.8, 11.3)		6.3 (4.9, 7.8)		6.3 (5.3, 7.3)	
Not answered	8.2 (6.6, 9.8)		5.6 (4.3, 7.0)		5.7 (4.7, 6.6)	
Total	100	100	100	100	100	100
Heroin						
*n*	1103	940	1106	991	2309	2068
No action	1.5 (0.7, 2.2)	1.7 (0.9, 2.5)	3.8 (2.7, 4.9)	4.2 (3.0, 5.5)	2.8 (2.1, 3.4)	3.1 (2.3, 3.8)
Caution or warning	2.4 (1.5, 3.4)	2.9 (1.8, 3.9)	3.3 (2.2, 4.3)	3.6 (2.5, 4.8)	2.7 (2.1, 3.4)	3.0 (2.3, 3.8)
Referral—education	13.8 (11.7, 15.8)	16.2 (13.8, 18.5)	16.5 (14.4, 18.7)	18.5 (16.1, 20.9)	15.6 (14.1, 17.1)	17.4 (15.8, 19.0)
Referral—treatment	25.9 (23.3, 28.5)	30.4 (27.5, 33.4)	35.1 (32.3, 37.9)	39.2 (36.1, 42.2)	32.9 (31.0, 34.8)	36.7 (34.6, 38.8)
Fine $200	2.1 (1.2, 2.9)	2.4 (1.5, 3.4)	1.7 (1.0, 2.5)	1.9 (1.1, 2.8)	2.2 (1.6, 2.8)	2.4 (1.8, 3.1)
Fine $1000	14.1 (12.0, 16.1)	16.5 (14.1, 18.9)	9.2 (7.5, 10.9)	10.3 (8.4, 12.2)	10.6 (9.4, 11.9)	11.8 (10.5, 13.2)
Community service order	4.1 (2.9, 5.2)	4.8 (3.4, 6.2)	4.0 (2.8, 5.1)	4.4 (3.2, 5.7)	4.3 (3.5, 5.1)	4.8 (3.9, 5.7)
Weekend detention	3.8 (2.7, 4.9)	4.5 (3.1, 5.8)	2.9 (1.9, 3.9)	3.2 (2.1, 4.3)	3.0 (2.3, 3.7)	3.4 (2.6, 4.2)
Prison	16.7 (14.5, 18.9)	19.6 (17.0, 22.1)	12.1 (10.2, 14.0)	13.5 (11.4, 15.7)	14.2 (12.7, 15.6)	15.8 (14.2, 17.4)
Other	0.9 (0.3, 1.5)	1.1 (0.4, 1.7)	1.0 (0.4, 1.6)	1.1 (0.5, 1.8)	1.3 (0.9, 1.8)	1.5 (1.0, 2.0)
Don't know	7.3 (5.7, 8.8)		5.2 (3.9, 6.5)		5.1 (4.2, 6.0)	
Not answered	7.5 (6.0, 9.1)		5.2 (3.9, 6.6)		5.3 (4.4, 6.2)	
Total	100	100	100	100	100	100
Methamphetamine						
*n*	1103	909	1105	962	2308	2023
No action	1.5 (0.7, 2.2)	1.8 (0.9, 2.6)	4.0 (2.8, 5.1)	4.6 (3.3, 5.9)	2.7 (2.1, 3.4)	3.1 (2.4, 3.9)
Caution or warning	2.8 (1.8, 3.8)	3.4 (2.2, 4.6)	3.9 (2.8, 5.0)	4.5 (3.2, 5.8)	3.0 (2.3, 3.7)	3.4 (2.6, 4.2)
Referral—education	13.1 (11.1, 15.0)	15.8 (13.5, 18.2)	15.5 (13.3, 17.6)	17.8 (15.4, 20.2)	14.2 (12.8, 15.6)	16.2 (14.6, 17.8)
Referral—treatment	24.9 (22.4, 27.5)	30.3 (27.3, 33.2)	33.3 (30.5, 36.1)	38.3 (35.2, 41.3)	31.5 (29.6, 33.4)	36.0 (33.9, 38.1)
Fine $200	2.2 (1.3, 3.0)	2.6 (1.6, 3.7)	2.3 (1.4, 3.1)	2.6 (1.6, 3.6)	2.7 (2.0, 3.3)	3.1 (2.3, 3.8)
Fine $1000	13.3 (11.3, 15.3)	16.2 (13.8, 18.6)	8.6 (6.9, 10.3)	9.9 (8.0, 11.8)	9.4 (8.3, 10.6)	10.8 (9.4, 12.1)
Community service order	3.8 (2.7, 4.9)	4.6 (3.3, 6.0)	3.4 (2.4, 4.5)	4.0 (2.7, 5.2)	4.0 (3.2, 4.8)	4.6 (3.7, 5.5)
Weekend detention	3.4 (2.3, 4.4)	4.1 (2.8, 5.4)	3.5 (2.4, 4.6)	4.1 (2.8, 5.3)	3.6 (2.9, 4.4)	4.2 (3.3, 5.0)
Prison	16.1 (14.0, 18.3)	19.6 (17.0, 22.2)	11.4 (9.5, 13.3)	13.1 (11.0, 15.2)	15.0 (13.6, 16.5)	17.2 (15.5, 18.8)
Other	1.4 (0.7, 2.0)	1.7 (0.8, 2.5)	1.2 (0.5, 1.8)	1.4 (0.6, 2.1)	1.3 (0.9, 1.8)	1.5 (1.0, 2.1)
Don't know	9.9 (8.1, 11.6)		7.3 (5.8, 8.9)		6.7 (5.7, 7.7)	
Not answered	7.7 (6.1, 9.3)		5.6 (4.3, 7.0)		5.7 (4,7, 6.6)	
Total	100	100	100	100	100	100
Hallucinogens						
*n*	1103	870	1106	947	2308	1982
No action	3.4 (2.4, 4.5)	4.4 (3.0, 5.7)	12.8 (10.9, 14.8)	15.0 (12.7, 17.3)	9.8 (8.6, 11.1)	11.5 (10.1, 12.9)
Caution or warning	6.2 (4.7, 7.6)	7.8 (6.0, 9.6)	10.6 (8.8, 12.4)	12.4 (10.3, 14.5)	8.9 (7.7, 10.0)	10.3 (9.0, 11.7)
Referral—education	16.1 (14.0, 18.3)	20.5 (17.8, 23.1)	16.9 (14.7, 19.1)	19.7 (17.2, 22.3)	17.2 (15.7, 18.8)	20.1 (18.3, 21.8)
Referral—treatment	18.8 (16.5, 21.1)	23.8 (21.0, 26.6)	18.9 (16.6, 21.2)	22.1 (19.4, 24.7)	19.3 (17.7, 20.9)	22.5 (20.6, 24.3)
Fine $200	4.1 (2.9, 5.2)	5.2 (3.7, 6.6)	4.2 (3.0, 5.3)	4.9 (3.5, 6.2)	4.9 (4.0, 5.8)	5.7 (4.7, 6.7)
Fine $1000	11.3 (9.5, 13.2)	14.4 (12.0, 16.7)	8.8 (7.1, 10.4)	10.2 (8.3, 12.2)	8.9 (7.7, 10.0)	10.3 (9.0, 11.7)
Community service order	2.7 (1.8, 3.7)	3.4 (2.2, 4.7)	3.1 (2.1, 4.1)	3.6 (2.4, 4.8)	3.8 (3.0, 4.5)	4.4 (3.5, 5.3)
Weekend detention	3.2 (2.1, 4.2)	4.0 (2.7, 5.3)	2.4 (1.5, 3.4)	2.9 (1.8, 3.9)	2.8 (2.1, 3.5)	3.3 (2.5, 4.1)
Prison	11.5 (9.6, 13.4)	14.6 (12.3, 16.9)	7.1 (5.5, 8.6)	8.2 (6.5, 10.0)	9.0 (7.8, 10.2)	10.5 (9.1, 11.8)
Other	1.5 (0.8, 2.3)	2.0 (1.0, 2.9)	0.9 (0.3, 1.5)	1.1 (0.4, 1.7)	1.3 (0.8, 1.7)	1.5 (0.9, 2.0)
Don't know	12.8 (10.8, 14.8)		8.4 (6.8, 10.0)		8.1 (7.0, 9.2)	
Not answered	8.3 (6.7, 10.0)		6.0 (4.6, 7.4)		6.0 (5.1, 7.0)	
Total	100	100	100	100	100	100

*Note*: 95% confidence intervals reported in brackets.

Abbreviation: NSW, New South Wales.

**TABLE A3:** Choice of criminal penalties for possession for personal use of Cannabis, by region, 2022/2023.

	Including ‘unsure/don't know’				Excluding ‘unsure/don't know’		
	*n*	Retain %	Remove %	Unsure/don't know %	*n*	Retain %	Remove %
GWS	1104	23.7 (21.2, 26.2)	52.7 (49.8, 55.7)	23.6 (21.0, 26.1)	844	31.0 (27.9, 34.2)	69.0 (65.8, 72.1)
Rest of Sydney	1106	13.3 (11.3, 15.3)	70.3 (67.6, 72.9)	16.5 (14.3, 18.6)	924	15.9 (13.6, 18.3)	84.1 (81.7, 86.4)
Rest of NSW	2309	15.3 (13.8, 16.8)	69.4 (67.5, 71.3)	15.3 (13.8, 16.8)	1956	18.0 (16.3, 19.8)	82.0 (80.2, 83.7)

*Note*: 95% confidence intervals reported in brackets.

Abbreviations: GWS, Greater Western Sydney; NSW, New South Wales.
